# Structural and Functional Characterization of OXA-48: Insight into Mechanism and Structural Basis of Substrate Recognition and Specificity

**DOI:** 10.3390/ijms222111480

**Published:** 2021-10-25

**Authors:** Jiachi Chiou, Qipeng Cheng, Perry Tim-fat Shum, Marcus Ho-yin Wong, Edward Wai-chi Chan, Sheng Chen

**Affiliations:** 1 State Key Laboratory of Chiroscience, Department of Applied Biology and Chemical Technology, The Hong Kong Polytechnic University, Hung Hom, Kowloon, Hong Kong, China; jiachi.amber.chiou@polyu.edu.hk (J.C.); peter-qipeng.cheng@connect.polyu.hk (Q.C.); tim-fat-perry.shum@connect.polyu.hk (P.T.-f.S.); marcus-ho-yin.wong@polyu.edu.hk (M.H.-y.W.); edwardwchan@yahoo.com.hk (E.W.-c.C.); 2 Department of Infectious Diseases and Public Health, Jockey Club College of Veterinary Medicine and Life Sciences, City University of Hong Kong, Kowloon, Hong Kong, China

**Keywords:** carbapenemase, OXA-48, active site residues, interaction, β-lactams

## Abstract

Class D β-lactamase OXA-48 is widely distributed among Gram-negative bacteria and is an important determinant of resistance to the last-resort carbapenems. Nevertheless, the detailed mechanism by which this β-lactamase hydrolyzes its substrates remains poorly understood. In this study, the complex structures of OXA-48 and various β-lactams were modeled and the potential active site residues that may interact with various β-lactams were identified and characterized to elucidate their roles in OXA-48 substrate recognition. Four residues, namely S^70^, K^73^, S^118^, and K^208^ were found to be essential for OXA-48 to undergo catalytic hydrolysis of various penicillins and carbapenems both in vivo and in vitro. T^209^ was found to be important for hydrolysis of imipenem, whereas R^250^ played a major role in hydrolyzing ampicillin, imipenem, and meropenem most likely by forming a H-bond or salt-bridge between the side chain of these two residues and the carboxylate oxygen ions of the substrates. Analysis of the effect of substitution of alanine in two residues, W^105^ and L^158^, revealed their roles in mediating the activity of OXA-48. Our data show that these residues most likely undergo hydrophobic interaction with the R groups and the core structure of the β-lactam ring in penicillins and the carbapenems, respectively. Unlike OXA-58, mass spectrometry suggested a loss of the C6-hydroxyethyl group during hydrolysis of meropenem by OXA-48, which has never been demonstrated in Class D carbapenemases. Findings in this study provide comprehensive knowledge of the mechanism of the substrate recognition and catalysis of OXA-type β-lactamases.

## 1. Introduction

OXA-type β-lactamases belong to class D β-lactamases (DBLs) which are responsible for resistance to β-lactam antibiotics [1]. Most of the OXA β-lactamases were identified as plasmid-encoded β-lactamases in Gram-negative bacteria until the discovery of *Acinetobacter baumannii* strains harboring chromosomally encoded OXA-51-like OXA β-lactamases, suggesting these genes are intrinsically located in the *Acinetobacter baumannii* -chromosome and play a role in resistance development [2]. Unlike classes A and C β-lactamases that have been well documented with activities towards penicillins, cephalosporins, and carbapenems, OXA β-lactamases were initially identified as penicillinases with higher activity on oxacillin, and were hence assigned the prefix of OXA. Some OXA variants were later found to be able to hydrolyze oxyimino-cephalosporins and carbapenems; such variants were therefore regarded as extended-spectrum β-lactamases (ESBL) and carbapenem-hydrolyzing class D β-lactamases (CHDLs), respectively [3,4,5]. In particular, the CHDLs have become an emerging threat to clinical treatment, as carbapenems are the last resort agents used in treatment of infections by multidrug resistant bacteria. The emergence of CHDLs in the Enterobacteriaceae, particularly *Klebsiella* spp., is a major clinical concern [6].

Based on amino acid sequence identity, CHDLs have been subdivided into several subgroups, among which OXA-23, OXA-24/40, OXA-51, OXA-58, and OXA-48-like carbapenemases are both identified in chromosome and mobile elements of carbapenem-resistant strains [7,8]. Moreover, OXA-48-like carbapenemases represent a significant concern considering that they are often plasmid-borne with IS elements, which facilitate their spread among members of Enterobacteriaceae [9,10]. In the past decade, OXA-48 type carbapenemases have gradually become prevalent in certain parts of the world (e.g., the Middle East, North Africa, and European countries), posing severe challenge to global effort to control antimicrobial resistance [11]. Like other DBLs, OXA-48 harbors three highly conserved motifs: (I) S^70^-X-X-K^73^, where X represents a variable residue; (II) S^118^-X-V/I, which is equivalent to the S^130^-D-N motif in class A β-lactamases and Y-A/S/N in AmpC β-lactamases and (III) K^208^-T-G [4,12]. OXA-48 also has two other conserved motifs including Y/F^144^-G-N and W^221^-X-X-G that only exist in class D β-lactamases [13,14].

Typical hydrolysis of β-lactam antibiotics by serine β-lactamases follows an acylation-deacylation process. It is generally accepted that class A β-lactamases employ S^70^ as the nucleophile in the acylation reaction with help from K^73^, which facilitates the protonation of the β-lactam nitrogen in the acylation step and the protonation of S^70^-Oγ diacylation [15,16,17]. Unlike classes A and C β-lactamases, the substrate hydrolysis mechanism of DBLs is less understood. It has been proposed that DBLs use S^70^ as the nucleophile in the acylation process with help from K^73^ that acts as the general base during both acylation (deprotonating S^70^) and deacylation steps (deprotonating the catalytic water molecule) [18]. During this process, the K^73^ of OXA variants may undergo post-translational modification and become carbamylated K^73^ (KCX^73^), which then plays a similar role as E^166^ in ABLs [19,20]. Extensive structure-function studies have been performed on the active site residues in narrow-spectrum DBLs, yet the functional roles of residues in the active pocket of OXA-48 carbapenemase in substrate recognition and catalysis have never been demonstrated. In this study, we characterize the key residues in the active site of OXA-48, in order to provide a comprehensive understanding of the roles of these residues in substrate recognition and catalysis, using an approach used for mechanistic study of other OXA-type β-lactamases.  

## 2. Results and Discussion

### 2.1. Effect of NaHCO_3_ on the Activity of OXA-48

**Figure 1:**
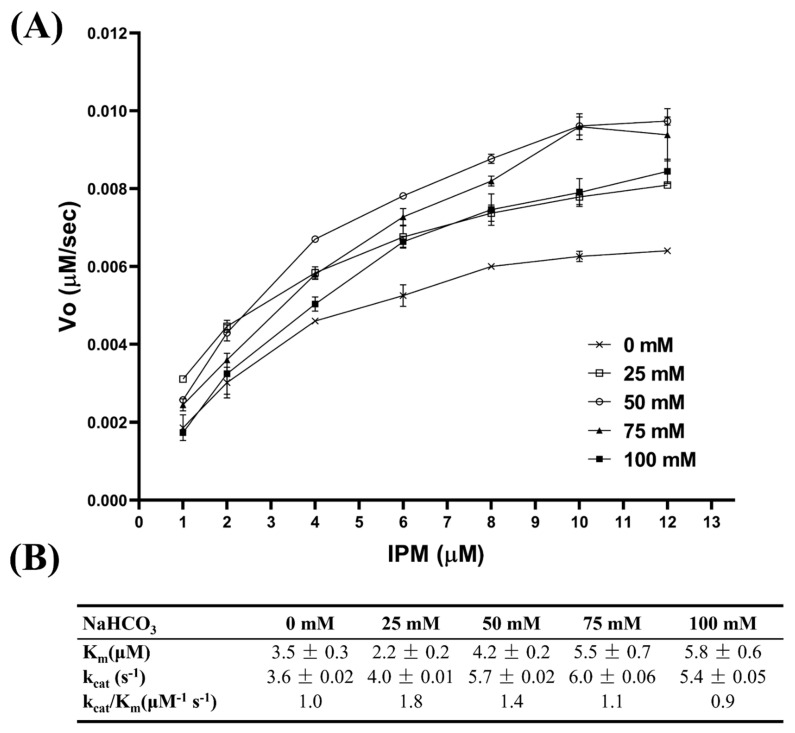
Effect of NaHCO_3_ on the initial rate (**A**) and kinetic parameters (**B**) of OXA−48 on imipenem (IPM) hydrolysis.

To minimize the effect of His_6_-tag on the activity of OXA-48, thrombin was used to remove the His6-tag from all mOXA-48 and its derivatives characterized in this work. NaHCO_3_ provided the necessary amount of CO_2_ to form N-carboxylation of K^73^, which plays a key role in the catalysis mechanism of OXA-10 [21]. The initial rate of OXA-48-mediated imipenem hydrolysis increased in the presence of 25 mM or 50 mM of NaHCO_3_, with comparable catalytic efficiency (*k_cat_*/*K_m_*) (Figure 1). OXA-48 exhibited high activity on nitrocefin in the presence of 25 mM or 50 mM sodium bicarbonate in a previous study [22]. The catalytic efficiencies, as represented as *k_cat_*/*K_m_*, gradually dropped when the concentration of NaHCO_3_ was increased to 75 and 100 mM. A concentration of 50 mM for NaHCO3 is probably saturating; a higher concentration of sodium bicarbonate might inhibit the activity of OXA-48. Measuring the slopes to determine the initial rates of imipenem hydrolysis in the presence of 25 mM NaHCO_3_ was considered unreliable. Hence 50 mM of NaHCO_3_ was used in the assay buffer for all kinetic assays in this study, which is consistent with that used in previous reports [20,22].

### 2.2. Residues Involved in the Hydrolysis of β-Lactam Ring

**Figure 2:**
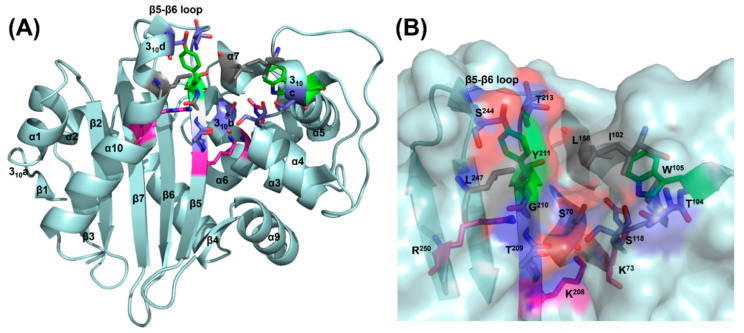
Potential residues that may be involved in substrate recognition and catalysis of OXA-48. (**A**) The secondary structures and motifs; (**B**) the important residues in the active pocket studied in this work are shown as gray (hydrophobic a.a.), green (aromatic a.a.), magenta (basic a.a.) and purple (nucleophilic a.a.) sticks. Amino acid: a.a.

The active cleft of OXA-48 is composed of several secondary structures including 310c, α7 and α10 helixes, motifs I (helix 310b), II (α4-α5 loop), III (β5) and β5-β6 loop [23]. Several residues including S^70^, K^73^ (located in motif I), S^118^ (motif II), K^208^, T^209^, G^210^, Y^211^ (motif III) and R^250^ (α10) of OXA-48 in the active sites were substituted by alanine to assess their role in conferring catalytic activities towards several β-lactam antibiotics (Figure 2). Substitution of alanine in the sites S^70^, K^73^, S^118^ and K^208^ resulted in dramatic reduction in MICs towards all penicillins and carbapenems tested (Table 1). The mutations which resulted in the S^70^A and K^73^A changes in OXA-48 were too inactive for determination of kinetic constants, whereas the S^118^A and K^208^A substitutions exhibited severely reduced catalytic efficiencies, with a five to ten-fold higher *K*_m_ and much lower *k*_cat_ towards ampicillin, carbenicillin, piperacillin, oxacillin, imipenem and meropenem when compared to the wild-type OXA-48 enzyme (Table 2). The impact of the S^70^A and K^73^A substitutions were expected as these two residues have been proposed to serve roles as the active site serine and the general base for acylation and deacylation, respectively. The role of S^70^ and K^73^ have were investigated in Schneider’s study, in which the S^70^G (DBL standard numbering) change in OXA-1 resulted in formation of an enzyme that still bound to the substrate but was unable to form a covalent acyl-enzyme intermediate, while substitution of carboxylated-K^73^ allowed the enzyme to form an acyl-enzyme intermediate but became unable to undergo diacylation [24]. The overall reduced activities of S^118^A and K^208^A both in vivo and in vitro towards all the β-lactam antibiotics tested in this study suggested that they may play similar roles as their counterparts in the class A β-lactamases, in which S^118^ donates a proton to the β-lactam nitrogen to facilitate acylation, and is stabilized by K^208^ [15,25].

**Figure 3:**
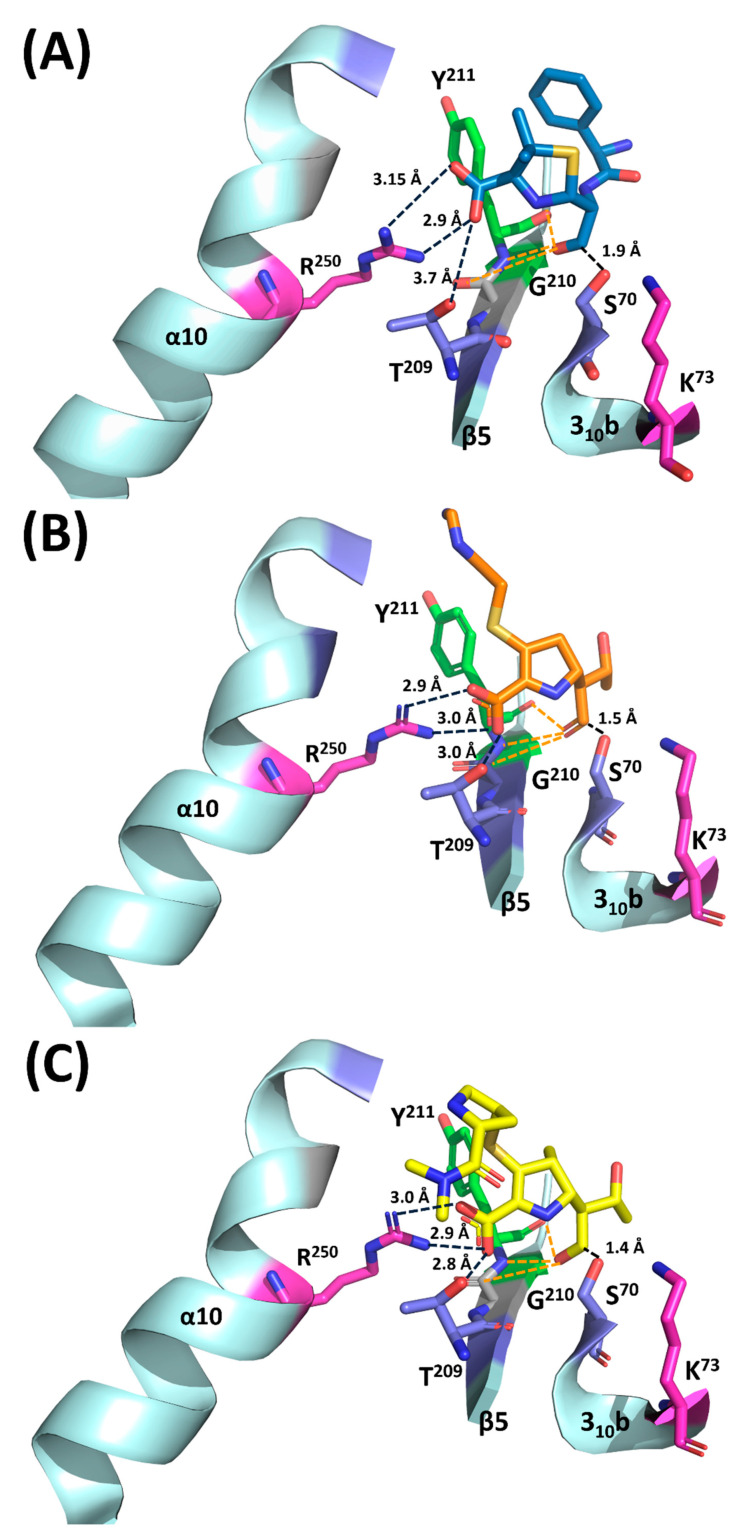
Interactions between ampicillin, imipenem, meropenem and the active site of OXA-48. (**A**) Modeling of ampicillin in the substrate pocket of OXA-48; ampicillin is depicted as blue stick. (**B**) Imipenem bound to OXA-48 in the substrate pocket (6P97); imipenem is depicted as orange stick. (**C**) Meropenem bound to OXA-48 in the substrate pocket (6P98); meropenem is depicted as yellow stick. Potential H-bonds between the residues of OXA-48 and carboxylate oxygens of the original β-lactam ring (orange) and that of the fused ring (black) are shown as dash lines.

The mutation associated with the T^209^A change causes significant decreases in MICs towards imipenem, but not the other β-lactams (Table 1). Consistently, the kinetic constants of enzyme harboring the T^209^A substitution exhibited 1.5 to 8-folds reduction in catalytic efficiencies (*k_cat_*/*K_m_*) against ampicillin, carbenicillin, piperacillin, oxacillin and meropenem, as well as an approximately 833-fold reduction in catalytic efficiency against imipenem (Table 2). Superposition of the OXA-48 structure with three complex structures of the OXA-type enzymes and ampicillin/imipenem revealed that T^209^-Oγ2 may form a moderate H-bond (3.0 Å or 2.8 Å) with the carboxylate oxygen of imipenem or meropenem, respectively. A weak contact with the carboxylate oxygen of ampicillin at a distance of about 3.7 Ǻ may explain the low ability of the enzyme in carrying the T^209^A substitution in hydrolyzing ampicillin (Figure 3).

R^250^ of OXA-48 appeared to be more important for the hydrolysis of imipenem and meropenem than penicillins. The R^250^A substitution exhibited a two to eight-fold reduction in MICs towards ampicillin, amoxicillin, carbenicillin and piperacillin, and a ≥16-fold reduction in MICs towards imipenem and meropenem (Table 1). Similarly, the kinetic assay of the effect of the R^250^A change exhibited more significant reduction in catalytic hydrolysis of these two carbapenems when compared to the penicillins (Table 2). Analysis of the OXA-48-imipenem structure and the OXA-48-meropenm structure revealed the presence of strong H-bonds between R^250^-Nζ and the carboxylate oxygen ions of imipenem or meropenem, with distances of 2.9 -3.0 Å or 2.9 -3.0 Å, respectively (Figure 3). A relatively long distance between R^250^-Nζ and the carboxylate oxygen of ampicillin was observed from the model structure of OXA-48-ampicillin (Figure 3). Our biochemical data regarding the effects of the R^250^A substitution on hydrolyzing penicillins and carbapenems were consistent with the modelled complex structure of OXA-48 and various β-lactams.

Our data showed that the G^210^A and Y^211^A changes did not have significant effects on the hydrolysis of β-lactams. Analysis of the OXA-48-amipicllin, OXA-48-imipenem and OXA-48-meropenem complexes showed that potential connections could be formed between the peptide (main chain) oxygen or nitrogen of Y^211^, as well as the newly formed carboxylate oxygen from original carbonyl group of β-lactams (Figure 3). Similarly, structures also suggested that potential van der Waals bonds could be formed between the peptide oxygen of G^210^ and one of the carboxylate ions of meropenem and imipenem. These features were also observable in other meropenem and imipenem complex structures [26,27,28]. Alanine substitution at Y^211^ and G^210^ could still maintain the peptide oxygen and nitrogen, which may explain the limited or zero effect of G^210^A and Y^211^A mutations of OXA-48 on the MICs towards all the β-lactams [19,26,29,30].

### 2.3. Residues Involved in Interaction between the R Groups of β-Lactam Antibiotics

Analysis of the complex structures of OXA-48 and different β-lactam antibiotics (ampicillin, meropenem, imipenem, and doripenem) predicted that several residues, including I^102^, T^104^, W^105^ (located at 310c), L^158^ (α7), T^213^ (β5-β6 loop), S^244^ (helix 310b) and L^247^ (α10), may potentially facilitate binding to R groups of substrates in the active site of OXA-48 (Figure 2); such interaction was also observed in previous structures [26,27,31]. Different from their counterpart residues, Y^112^ and M^223^, which form the ceiling of the active pocket in OXA-24, I^102^ and T^213^ of OXA-48 were located in the opposite site on top of the active cleft and did not form a roof (Figure 2). I^102^A exhibited limited or no effect on the MIC of all the penicillins and carbapenems, while T^213^A caused four to eight-fold reductions of MIC against ampicillin, amoxicillin, piperacillin, imipenem and meropenem (Table 2). The modeling results suggest the T^213^ side chain is in close proximity of the R group of ampicillins, but much farther from the R groups of imipenem and meropenem. A previous study suggested the importance of the β5-β6 loop, where T^213^ is located, on the carbapenemase activities of DBLs. In such previous study the non-carbapenemase OXA-10 turned into a carbapenemase with slight increase in MIC and kinetic constants against ertapenem and imipenem, respectively, after being introduced with the loop between β5-β6 from OXA-24 and OXA-48 [32]. These observations implied that T^213^A may cause subtle changes in this loop and hence attenuates the activity of OXA-48 on β-lactams concerned.

**Figure 4:**
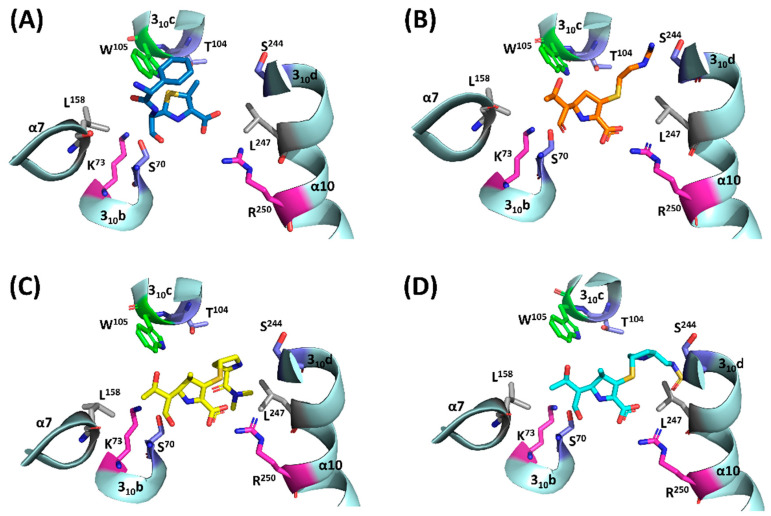
Overview of residues of OXA-48 involved in binding to different antibiotics. (**A**) Ampicillin (blue) was modelled in the active site of OXA-48, (**B**) imipenem (orange, 6P97) in the active site of OXA-48; (**C**) meropenem (yellow, 6P98) in the active site of OXA-48; (**D**) doripenem (cyan, 6P9C) in the active site of OXA-48.

**Figure 5:**
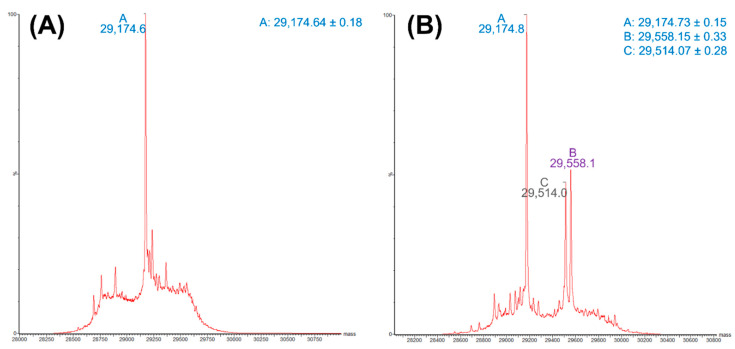
Analysis of interaction between OXA-48 and meropenem by ESI-MS: (**A**) mass spectrum of denatured OXA-48, mass at 29,174.6 Da; (**B**) mass spectrum of denatured OXA-48 after incubation with meropenem, apo OXA-48 (29,174.8 Da), and two meropenem binding product (29,514.0 Da and 29,558.1 Da).

Both W^105^A and L^158^A showed reduced MICs against ampicillin, amoxicillin, piperacillin, oxacillin, imipenem and meropenem, with L^158^A exhibiting a stronger impact (Table 1). The kinetic parameters of these two residues exhibited reduced catalytic efficiencies to the six substrates tested, with the highest reduction of *k_cat_*/*K_m_* against imipenem (Table 2). L^158^A showed more significant reduction in *k_cat_*/*K_m_* against ampicillin, carbenicillin, pipercillin and oxacillin, but not imipenem and meropenem, when compared to W^105^A; such a finding was consistent with the MIC results. In the structures of OXA-48 with several β-lactam antibiotics, W^105^ was suggested to interact with the R groups of ampicillin, imipenem, meropenem and doripenem, most likely through hydrophobic interaction [27,28,31]. Similarly, L^158^ was found to exhibit potential hydrophobic interaction with the core structure of penicillins and carbapenems, namely, the β-lactam ring and the fused ring (Figure 4). These observations were somewhat consistent with the results of MIC and kinetic assays in this work. Mass spectrometry of coincubation of OXA-48 and meropenem revealed three major complexes corresponding to the OXA-48 protein alone (29174.73 Da), OXA-48 with meropenem (29558.15 Da), and OXA-48 binding partially to meropenem, with a loss of 44 Da (29514.07 Da) (Figure 5). The findings suggest occurrence of a retro-aldol reaction which leads to elimination of the C6-hydroxyethyl group of meropenem during catalysis of OXA-48. Similar results have been shown in class A β-lactamase (BlaC) and Class C β-lactamases (ADC-7, CMY-2 and CMY-32), but it has never been observed in Class D β-lactamases [33,34,35]. On the other hand, two major products, namely a hydrolysis product and a lactone product, were detected by NMR upon incubation of OXA-48 with ertapenem, suggesting that the enzyme adopted a new catalytic mechanism without hydrolytic water [36]. The loss of CO_2_ (44 *m/z*) in the site of carboxylation of K^73^ in the OXA-48 might be another result of mass change. This phenomenon, which indicates that there is an OXA-48 apo enzyme, an OXA-48-meropenem complex, and an OXA-48 (the carboxylation of K^73^)-meropenem complex in the reaction system, needs to be confirmed in a future study.

### 2.4. Mechanisms of Substrate Recognition and Specificity by OXA-48

Different from OXA-24 that shows similar turnover rates for imipenem and meropenem, OXA-48 exhibited higher *k_cat_*/*K_m_* towards imipenem when compared to meropenem. This finding is consistent with a previous study which showed that OXA-48 exhibited strong preference for imipenem and panipenem over meropenem and ertapenem [23]. Docquier et al. also predicted that four residues, including H^109^, T^213^, R^214^ and S^244^ (DBL standard numbering) might be responsible for carbapenemase activity of OXA-48. Although T^213^A exhibited attenuated MICs towards imipenem and meropenem, this mutant still maintained mild carbapenemase activity. Substitution by alanine in S^244^ had no or limited effect on the MICs towards all the β-lactam antibiotics tested (Table 2), suggesting that this residue has little effect on the carbapenemase activity of OXA-48. The active site of OXA-48 exhibits higher similarity to OXA-1/10/13, with a more open active cleft when compared to OXA-23/24, as well as a roof composed of Y^112^ and M^223^ that exhibited the carbapenemase activity on only the latter two enzymes. Interestingly, OXA-48 acquired carbapenemase activity without losing activity towards oxacillin, which was poorly hydrolyzed by OXA-24 with a trade-off phenotype of becoming a carbapenemase. Different from the hydrolytic mechanism of OXA-58, the loss of the hydroxyethyl group in meropenem was observed in OXA-48, indicating that these two class D carbapenemases might adopt different hydrolytic mechanisms towards meropenem during the evolution process [5]. Furthermore, a carbamylated-K^73^ is required for the carbapenemase activity of OXA-48 and plays a key role in catalysis. The roles of S^70^ and carbamylated-K^73^ have been well illustrated in other OXA-type enzymes but not in OXA-48. This study demonstrated that at least four residues including S^70^, K^73^, S^118^ and K^208^, play critical roles in the substrate binding and catalysis of OXA-48, most likely through S^70^ and K^73^ which serve as the active site serine and general base for acylation and deacylation, respectively. Imipenem and meropenem in the structure of OXA-48 (K^73^A) adopted similar conformations when compared to the previous OXA-48 acyl-enzyme complexes, but the conformation of S^118^ was slightly altered because of elimination of a hydrogen bond between K^73^ and S^118^ [28]. S^118^ may play a role similar to its counterpart in class A β-lactamases, facilitating acylation by donating a proton β-lactam nitrogen, while K^208^ could help to stabilize this process. This finding was supported by the finding that S^118^G exhibited lower activity towards ampicillin and imipenem in Nesheim’s study [37]. T^209^ of OXA-48 may play a role in stabilizing the enzyme-substrate intermediate by forming a H-bond between T^209^-Oγ2 and one of the imipenem carboxylate oxygen ions, while R^250^ could interact with meropenem, imipenem and penicillins, likely by forming a H-bond between R^250^-Nζ and the carboxylate oxygen ions of these substrates. The importance of R250 has also been evaluated by analysis of a single mutant R^250^A [37]. W^105^ may help to localize the substrates, most likely by forming hydrophobic interactions with the R groups of penicillins, imipenem and meropenem, while L^158^ could form a hydrophobic interaction with the core structure of penicillins and carbapenems. Like OXA-1, OXA-10, OXA-24 and OXA-58, OXA-48 also adopts the carbamylated K^73^ for catalysis, during which the C6 hydroxyethyl group of carbapenem may be removed [38,39]. To summarize, this work represents a comprehensive study covering most of the critical residues that may affect substrate binding and catalysis in the active site of OXA-48. Our findings may shed light on the mechanism of the substrate specificity of OXA-48.

## 3. Materials and Methods

### 3.1. Antibiotics and Media

Amoxicillin, carbenicillin, imipenem and meropenem were purchased from Melonepharma Co. (Dalian, China). Piperacillin and oxacillin were purchased from Sigma Chemical Co. (St. Louis, MO, USA) while isopropyl β-D-1-thiogalactopyranoside (IPTG), ampicillin and kanamycin were purchased from IBI Inc (Boca Raton, FL, USA). Sodium hydrogen carbonate was purchased from Fisher Chemical (Waltham, MA, USA). Luria broth (LB) was purchased from BD Co (Franklin Lakes, NJ, USA) while Muller-Hinton broth (MHB) was purchased from OXOID Co (Hampshire, UK).

### 3.2. Recombinant OXA-48 and Site-Directed Mutagenesis

*bla*_OXA-48_ was synthesized by PCR-based amplification following procedures described in a previous study [40]. The correct sequence was further constructed in a pET28b plasmid and transformed into *E. coli* Tg1 in our laboratory. Two versions of the *bla*_OXA-48_ constructs were synthesized, which were the full-length gene (pET28-*bla*_OXA-48_) and a truncated gene encoding K^23^ to P^265^ as well as carrying an N-terminal His6 tag (pET28-*bla*_H6-mOXA-48_). Point mutations in *bla*_OXA-48_ were generated using the GENEART^®^ Site-Directed Mutagenesis kit (Invitrogen Co., NY, USA) and transformed into *E. coli* DH5α. The sequences were confirmed and the transformants were selected from an LB plate supplemented with 50 µg/mL of Kanamycin. *E. coli* BL21(DE3) was transformed with the clones carrying selected mutations for over-expression of the gene product and purification.

### 3.3. Expression and Purification of OXA-48 and Mutants

Protein expression and purification have been described previously [41]. Briefly, an overnight culture of *E. coli* carrying pET28-*bla*_H6-mOXA-48_ was subcultured into 500 mL LB and incubated at 16 °C overnight in the presence of 1 mM IPTG, starting with an OD_600_ of 0.6. The cells were disrupted using a French press and centrifuged at 39,100× *g* for 60 min at 4 °C. The supernatant was passed through a Ni-NTA column. The eluted fractions containing the target proteins were pooled and concentrated with an Amicon Ultra-15 (NMWL = 10,000) centrifugal filter device. The His_6_-mOXA-48 was exchanged into a PBS buffer and the His6 tag was cleaved by Thrombin from bovine plasma (Sigma, USA) according to the product guide. His6 tag and uncut His6-mOXA-48 were removed by reloading into a Ni-NTA column. mOXA-48 protein without His6 tag was further purified by gel filtration (Superdex 75). The purified protein was concentrated to 15–40 mg/mL and analyzed using 12% of Sodium Dodecyl Sulfate-Polyacrylamide gel electrophoresis (SDS-PAGE).

### 3.4. Antimicrobial Susceptibility Testing

Minimum inhibitory concentrations (MICs) of different β-lactams including ampicillin, carbenicillin, amoxicillin, piperacillin, oxacillin, meropenem, and imipenem were tested on *E. coli* BL21 (DE3) expressing the recombinant β-lactamases using the microdilution method according to the CLSI procedure [42]. In brief, the pET28 constructs containing full-length *bla*_OXA-48_ and its corresponding variants were grown on an MH agar plate, incubated at 37 °C overnight, and transferred to MH broth supplemented with a serial concentration of selected β-lactams and 0.5 mM IPTG, followed by incubation at 37 °C for 16–20 h. Antimicrobial susceptibility testing of transformants was interpreted according to the CLSI guidelines. Determination of MIC was repeated at least in triplicate for each antibiotic tested. *E. coli* strains (ATCC 25922) was used as quality control.

### 3.5. Effect of NaHCO_3_ on the Kinetic Parameters

The effect of NaHCO_3_ on the kinetic parameters of OXA-48 was determined by incubating the enzyme with different concentrations of imipenem in 500µL of assay buffer (50 mM phosphate buffer, pH 7.0) in the presence of 0, 25, 50, 75 and 100 mM of NaHCO_3_. The kinetic constants of OXA-48 and its mutants towards different β-lactams were determined as aforementioned except that the recipe of the assay buffer was 50 mM phosphate buffer, pH 7.0, 50 mM NaHCO_3_. The initial velocities of substrate hydrolysis for ampicillin (Δε_235_ = −820 M^−1^ cm^−1^), carbenicillin (Δε_235_ = −780 M^−1^ cm^−1^), piperacillin (Δε_235_ = −820 M^−1^ cm^−1^), oxacillin (Δε_260_ = −440 M^−1^ cm^−1^), imipenem (Δε_300_ = −9000 M^−1^ cm^−1^), and meropenem (Δε_300_ = −6500 M^−1^ cm^−1^) were measured by monitoring the changes in absorbance in a 1 cm quartz cuvette by a spectrometer (Perkin Elmer Lambda Bio20). The initial velocities versus substrate concentrations were measured and fitted to a nonlinear regression according to the Michaelis-Menten equation using GraphPad Prism8.0 (San Diego, CA, USA).

### 3.6. Structure Modelling and Analysis

Ampicillin was docked into the structure of OXA-48 and simulated using AutoDock 4.2 and its graphical user interface, ADT [43]. All calculations for docking were conducted by the Lamarckian Genetic Algorithm (LGA) method. The size of the grid box was 66 Å × 66 Å × 66 Å in the x, y, and z dimensions with a grid spacing of 0.375 Å. The energy scoring of grid maps was calculated using AutoGrid. All the docking parameters were set to their standard values. The docked model with the lowest docking energy and maximum docking number was chosen to represent its most favorable binding pose as predicted by the program. Complex structures of OXA-48-imipenem (6P97), OXA-48-meropenem (6P98), and OXA-48-doripenem (6P9C) were obtained from the PDB database [26]. Structures were analyzed and displayed by PyMOL [44].

### 3.7. Detection of Acyl-Enzyme Intermediate by ESI-TOF-MS

Electrospray ionization time-of-flight mass spectrometry (ESI-TOF-MS) was performed using a Waters Synapt G2-Si High-Definition Ion Mobility Mass Spectrometer to detect the acyl-enzyme complex. For the denaturing condition, a 100 µL enzyme-substrate reaction mixture in 50 mM ammonium bicarbonate (pH 8.0) was incubated at room temperature. At different time points, the reaction mixture was quenched, and the proteins were unfolded by adding an equal volume of 0.5% formic acid (*v/v*) in acetonitrile. The quenched reaction mixtures were then infused into the ESI source at a flow rate of 10 μL/min for TOF-MS analysis. Mass spectrometry was operated in positive ion mode with a *m/z* range of 100–2000 for detection of multiple charged proteins. The mass spectra obtained subsequently were deconvoluted by the Transform program (MassLynx 4.1, Waters).

## Figures and Tables

**Table 1:** MICs of OXA-48 constructs and variants towards different β-lactams.

	AMP ^a^	AMX	CAR	PIP	OXA	IPM	MEM
pET28	<1	2	4	0.5	8	<0.25	<0.004
WT OXA-48	512	512	256	4096	1024	2	1
S^70^A	<1	1	8	0.5	16	<0.25	<0.004
K^73^A	<1	2	8	0.5	16	<0.25	<0.004
S^118^A	<1	2	16	<0.25	16	<0.25	<0.004
K^208^A	<1	2	8	0.5	16	<0.25	<0.004
T^209^A	512	1024	256	2048	2048	<0.25	0.5
G^210^A	256	256	256	1024	2048	1	0.5
Y^211^A	512	256	256	2048	2048	1	0.5
R^250^A	64	128	128	1024	1024	<0.25	0.06
I^102^A	1024	512	256	2048	2048	1	1
T^104^A	512	512	256	1024	1024	1	1
W^105^A	128	128	256	1024	512	<0.25	0.125
L^158^A	64	64	128	128	256	<0.25	0.125
T^213^A	128	64	128	1024	1024	0.5	0.25
S^244^A	1024	512	256	2048	1024	2	1
L^247^A	2048	512	256	2048	2048	1	1

^a^ AMP, ampicillin; AMX, amoxicillin; CAR, carbenicillin; PIP, piperacillin; OXA, oxacillin; IPM, imipenem; MEM, meropenem.

**Table 2:** Kinetic constants of OXA-48 variants towards seven β-lactams.

	Constants	OXA-48 WT	S^118^A	K^208^A	T^209^A	R^250^A	W^105^A	L^158^A
AMP ^a^	*K_m_* (μM)	86 ± 6	>800	117 ± 8	125 ± 9	92 ± 7	81 ± 9	60 ± 11
	*k_cat_* (s^−1^)	817 ± 29	>10	3 ± 0.1	164 ± 5.8	234 ± 8.1	75 ± 3.7	20 ± 1.3
	*k_cat_*/*K_m_* (μM^−1^ s^−1^)	9.5	1.7 × 10^−2^	2.6 × 10^−2^	1.3	2.5	0.9	0.3
CAR	*K_m_* (μM)	57 ± 7	>500	421 ± 36	269 ± 23	147 ± 19	217 ± 12	79 ± 6
	*k_cat_* (s^−1^)	311 ± 22	>1	4 ± 0.2	323 ± 19	160 ± 12	127 ± 12	18 ± 0.7
	*k_cat_*/*K_m_* (μM^−1^ s^−1^)	5.5	6.6 × 10^−3^	9.5 × 10^−3^	1.2	1.1	0.6	0.2
PIP	*K_m_* (μM)	96 ± 10	>500	385 ± 12	93 ± 20	195 ± 35	162 ± 38	461 ± 91
	*k_cat_* (s^−1^)	197	>0.4	4 ± 0.1	61 ± 6.3	109 ± 12	58 ± 9.1	98 ± 14
	*k_cat_*/*K_m_* (μM^−1^ s^−1^)	2.1	3.0 × 10^−3^	1.0 × 10^−2^	0.7	0.6	0.4	0.2
OXA	*K_m_* (μM)	34 ± 3	337 ± 20	130 ± 11	81 ± 15	138 ± 13	116 ± 15	184 ± 11
	*k_cat_* (s^−1^)	162 ± 5.4	1.4 ± 0.1	2 ± 0.1	52 ± 4.6	94 ± 5.2	60 ± 4.4	20 ± 1.1
	*k_cat_*/*K_m_* (μM^−1^ s^−1^)	4.8	4.2 × 10^−3^	1.5 × 10^−2^	0.6	0.7	0.5	0.1
IPM	*K_m_* (μM)	4 ± 0.2	>150	>150	232 ± 87	926 ± 48	24 ± 2.9	>150
	*k_cat_* (s^−1^)	6 ± 0.02	>0.05	>0.005	0.4 ± 0.1	4 ± 1.9	0.03 ± 0.01	>1
	*k_cat_*/*K_m_* (μM^−1^ s^−1^)	1.5	7.1 × 10^−4^	3.0 × 10^−5^	1.8 × 10^−3^	2.2 × 10^−3^	1.3 × 10^−3^	1.0 × 10^−2^
MEM	*K_m_* (μM)	40 ± 0.3	>300	>300	60 ± 6.3	>300	65 ± 5.8	55 ± 4.9
	*k_cat_* (s^−1^)	0.1 ± 0.01	>0.02	>0.01	0.1 ± 0.01	>0.01	0.01 ± 0.001	0.04 ± 0.001
	*k_cat_*/*K_m_* (μM^−1^ s^−1^)	2.5 × 10^−3^	6.3 × 10^−5^	3.1 × 10^−5^	1.7 × 10^−3^	7.0 × 10^−5^	1.5 × 10^−4^	7.3 × 10^−4^

^a^ AMP, ampicillin; CAR, carbenicillin; PIP, piperacillin; OXA, oxacillin; IPM, imipenem; MEM, meropenem.

## Data Availability

Not applicable.
